# Manipulating Ce Valence in RE_2_Fe_14_B Tetragonal Compounds by La-Ce Co-doping: Resultant Crystallographic and Magnetic Anomaly

**DOI:** 10.1038/srep30194

**Published:** 2016-07-26

**Authors:** Jiaying Jin, Yujing Zhang, Guohua Bai, Zeyu Qian, Chen Wu, Tianyu Ma, Baogen Shen, Mi Yan

**Affiliations:** 1 School of Materials Science and Engineering, State Key Laboratory of Silicon Materials, Key Laboratory of Novel Materials for Information Technology of Zhejiang Province, Zhejiang University, Hangzhou 310027, China; 2 State Key Laboratory for Magnetism, Institute of Physics, Chinese Academy of Sciences, Beijing 100190, China

## Abstract

Abundant and low-cost Ce has attracted considerable interest as a prospective alternative for those critically relied Nd/Pr/Dy/Tb in the 2:14:1-type permanent magnets. The (Nd, Ce)_2_Fe_14_B compound with inferior intrinsic magnetic properties to Nd_2_Fe_14_B, however, cannot provide an equivalent magnetic performance. Since Ce valence is sensitive to local steric environment, manipulating it towards the favorable trivalent state provides a way to enhance the magnetic properties. Here we report that such a desirable Ce valence can be induced by La-Ce co-doping into [(Pr, Nd)_1−x_(La, Ce)_x_]_2.14_Fe_14_B (0 ≤ x ≤ 0.5) compounds via strip casting. As verified by X-ray photoelectron spectroscopy results, Ce valence shifts towards the magnetically favorable Ce^3+^ state in the composition range of x > 0.3, owing to the co-doping of large radius La^3+^ into 2:14:1 phase lattice. As a result, both crystallographic and magnetic anomalies are observed in the same vicinity of x = 0.3, above which lattice parameters *a* and *c*, and saturation magnetization *M*_*s*_ increase simultaneously. Over the whole doping range, 2:14:1 tetragonal structure forms and keeps stable even at 1250 K. This finding may shed light on obtaining a favorable Ce valence via La-Ce co-doping, thus maintaining the intrinsic magnetic properties of 2:14:1-type permanent magnets.

Larger energy product (*BH*)_max_ to reduce the size/weight of permanent magnets (PMs) has long been the goal of material scientists and engineers. An extraordinary progress occurred in 1980s with the discovery of Nd_2_Fe_14_B^1^, the strongest PM existing today, which has become an indispensable component of many mass-market consumer goods and industrial products. Under the new circumstance of global rare earth (RE) criticality, especially for those closely-relied REs Nd/Pr/Dy/Tb in the 2:14:1-type PMs, price-favorable and high-abundant La/Ce has triggered intense research as prospective alternatives^2^,^3^,^4^,^5^,^6^,^7^,^8^. The substitution of Nd with La and Ce in the tetragonal RE_2_Fe_14_B phase, however, is inevitably accompanied with magnetic dilution due to inferior intrinsic magnetic properties of (La, Ce)_2_Fe_14_B to Nd_2_Fe_14_B (room-temperature saturation magnetic polarization *J*_S_ and anisotropy field *H*_A_ for La_2_Fe_14_B/Ce_2_Fe_14_B/Nd_2_Fe_14_B are 1.38/1.17/1.60 T, and 20/26/73 kOe, respectively)^9^,^10^,^11^. Consequently, preparation of 2:14:1 tetragonal compounds with high La/Ce substitution and sustained intrinsic magnetic performance simultaneously is still a big challenge.

Since the La_2_Fe_14_B tetragonal structure is unstable^12^,^13^,^14^, the most abundant and cheapest Ce has attracted considerable interest for decades^15^,^16^,^17^,^18^,^19^,^20^,^21^,^22^,^23^. Different from Pr^3+^, Nd^3+^ and La^3+^ with stable valence, Ce generally exhibits a mixed valence of 3.44 due to the coexistence of trivalent 4f^1^ and tetravalent 4f^0^ electronic states in Ce_2_Fe_14_B^8^. Stabilizing the Ce^3+^ configuration with one localized 4f moment is beneficial for higher intrinsic magnetic properties, thus suppressing the magnetic dilution of Ce-doping RE_2_Fe_14_B compounds. Previous investigations on melt-spun ribbons^6^,^10^,^17^,^23^, hot-pressed^6^,^18^, die-upset^6^,^18^ and sintered magnets^19^,^20^ have revealed gradually decreased lattice constants of the 2:14:1 phase with increasing Ce content, which can be explained by the smaller lattice parameters of Ce_2_Fe_14_B compound (*a* = 8.76 Å and *c* = 12.11 Å) than those of Nd_2_Fe_14_B (*a* = 8.80 Å and *c* = 12.20 Å)^9^. Since the ion radius follows the relation *r* (Ce^3+^) > *r* (Nd^3+^) > *r* (Ce^4+^), the monotonic decreasing *a* and *c* implies that Ce valence keeps basically unchanged within the whole doping range, otherwise the lattice parameters will deviate from the monotonic variation.

Notably, the Ce valence is highly dependent on its steric environment. The Ce valence decreases with expanding site volume, which suggests the potential of tuning Ce valence via alloying^24^,^25^. For instance, Ce_2_Fe_14_B exhibits lattice expansion after hydriding, meanwhile the Ce valence shifts towards the moment-carrying 4f^1^ (+3) state compared to the unhydrided parent^24^,^25^. Calculations based on the density-functional theory also predict that La can function like interstitial hydrogen in the (La, Ce)_2_Fe_14_B compound to induce a favorable Ce^3+^ state^15^. Thus, if La, with largest atomic radius among all REs, co-dope with Ce into the (Nd, Pr)_2_Fe_14_B lattice, may be able to produce a similar crystal lattice expansion and induce a preferable Ce^3+^ configuration accordingly. In this work, we found that stable 2:14:1 tetragonal phase is formed in [(Pr, Nd)_1−x_(La, Ce)_x_]_2.14_Fe_14_B compounds even with *x* up to 0.5 by the commercialized strip casting technique for Nd-Fe-B sintered magnets^26^,^27^. XPS results verify a valence shift towards the favorable Ce^3+^ state merely in the composition range of x > 0.3. As a result, nonmonotonic dependences of lattice parameters *a*/*c* and saturation magnetization *M*_s_ on the La-Ce content x are observed simultaneously.

## Results and Discussions

**Figure 1:**
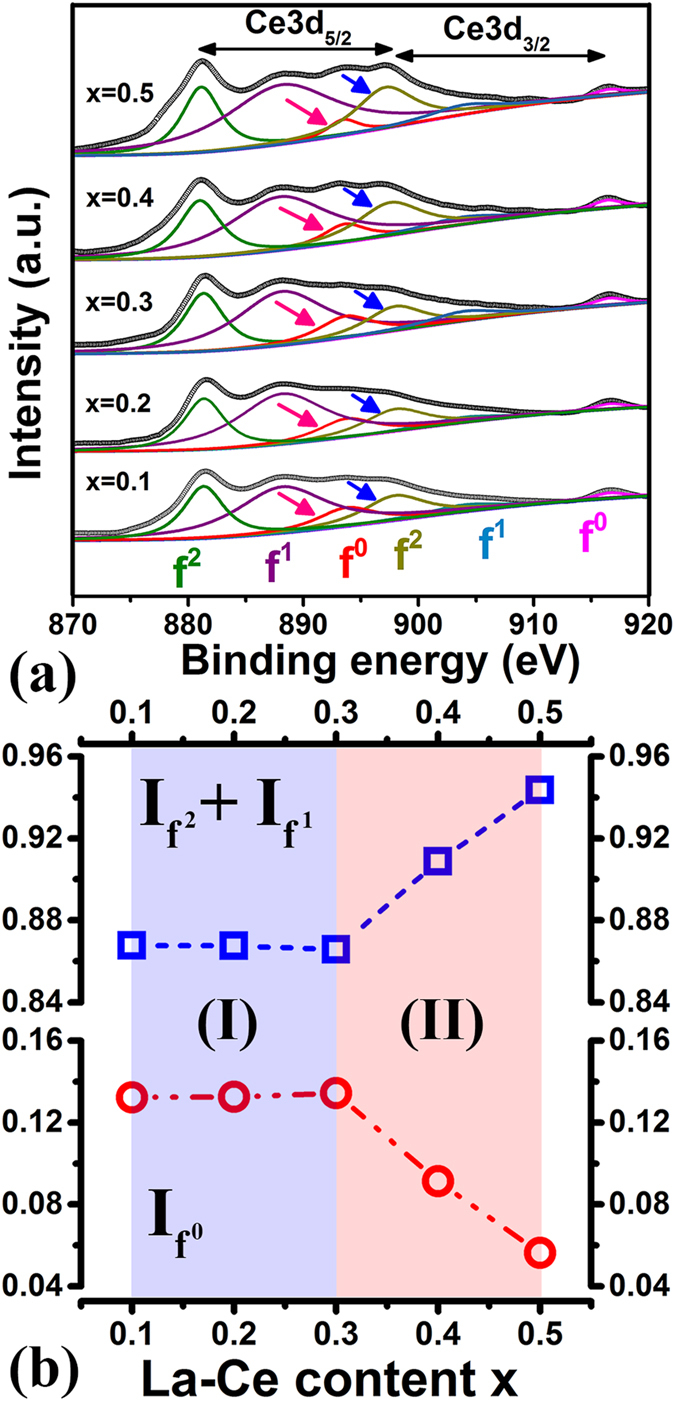
(**a**) XPS spectra of the Ce 3d level in [(Pr, Nd)_1−x_(La, Ce)_x_]_2.14_Fe_14_B strips with x = 0.1∼0.5. (**b**) The derived intensities of 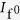 (circles in red color), and (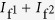) (squares in blue color) are plotted as a variation of La-Ce content x.

Figure 1 shows the Ce 3d spectra of [(Pr, Nd)_1−x_(La, Ce)_x_]_2.14_Fe_14_B (x = 0.1∼0.5) strips, suggesting that Ce valence changes with increasing La-Ce content. A Tougaard procedure^28^ is used to remove the background (blue color in Fig. 1a). When x ≤ 0.3 (as indicated by the composition range I in Fig. 1b), no obvious change in the Ce 3d spectra can be detected. Further increasing La-Ce content from 0.3 to 0.4 and 0.5 (composition range II), the peak intensity declines for Ce3d_5/2_ f^o^ lines (indicated by red arrows) and increases for the Ce3d_3/2_ f^2^ ones (indicated by blue arrows). The ratio *r*_0_ (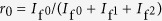) is calculated to evaluate the mixed valence of Ce, where 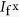 represents the weight of the f^x^ peak in the spectrum. As shown in Fig. 1b, where the 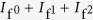 intensity is normalized to 1, 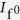 diminishes gradually from 0.1343 to 0.0913 and 0.0564 with x gradually increased from 0.3 to 0.4 and 0.5. This relatively lowered Ce^4+^ ratio (*r*_0_) with enhanced Ce^3+^ ratio reveals the shift of Ce valence towards the favorable Ce^3+^ state with 4f moment, verifying that La-Ce co-doping provides a way to manipulate the Ce valence by changing the La-Ce concentration.

**Figure 2:**
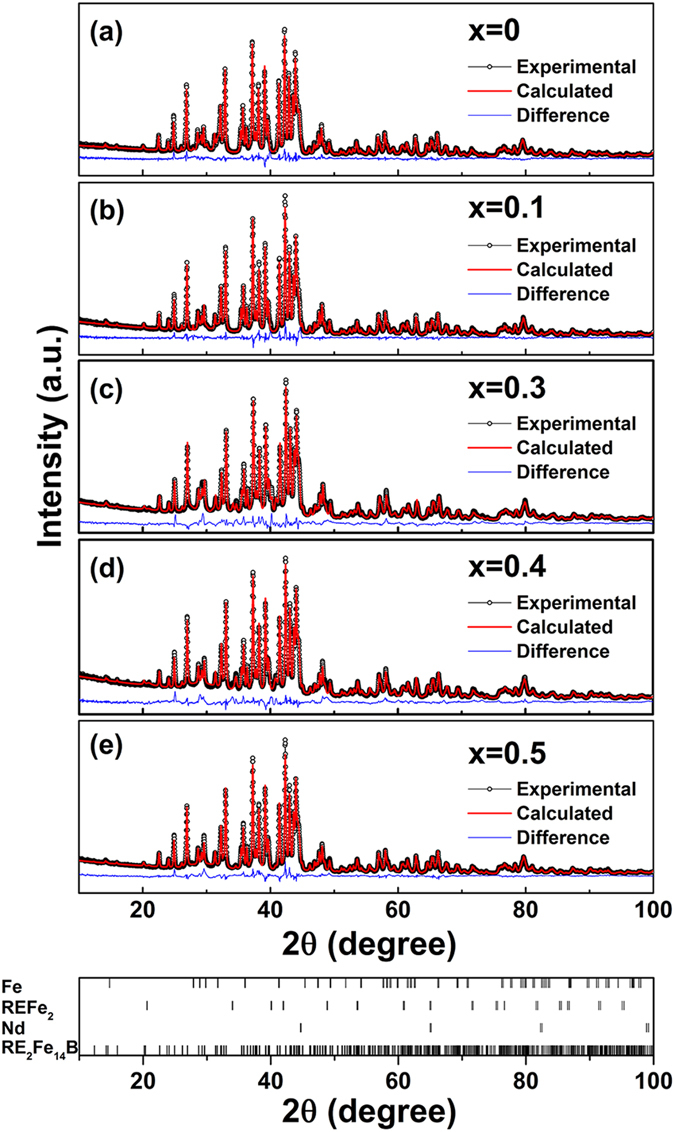
Rietveld refinement of step-scanned XRD patterns of [(Pr, Nd)_1−x_(La, Ce)_x_]_2.14_Fe_14_B powders for (**a**) x = 0, (**b**) x = 0.1, (**c**) x = 0.3, (**d**) x = 0.4, and (**e**) x = 0.5 at room temperature. Experimental pattern, calculated pattern, and their differences are given in black, red and blue colors, respectively. Bottom ticks mark the characteristic Bragg positions of RE_2_Fe_14_B, Nd, REFe_2_ and Fe phases, and serve as a guide to the eye.

**Figure 3:**
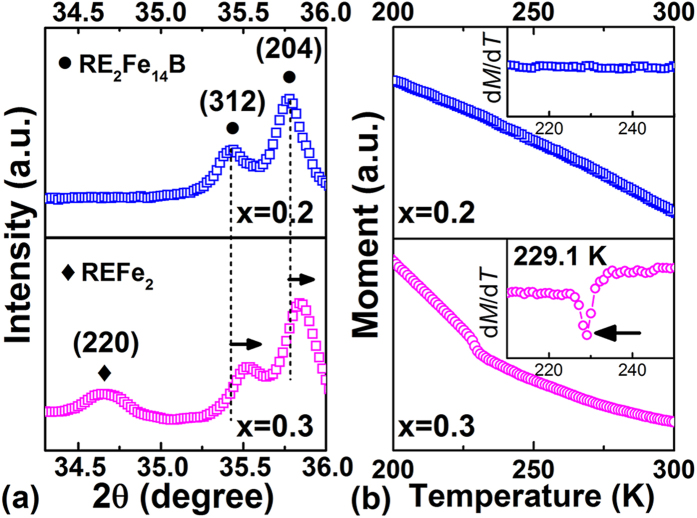
(**a**) Enlarged XRD patterns of 2θ between 34.3∼36° for samples (x = 0.2 and x = 0.3). (**b**) *M*-*T* curves for samples (x = 0.2 and x = 0.3) in the temperature range of 200∼300 K. Their corresponding d*M*/d*T*-*T* curves are shown in the top-right insets.

**Figure 4:**
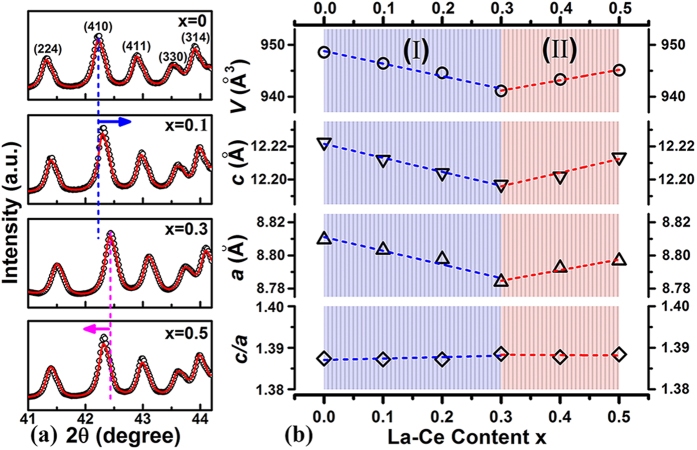
(**a**) Enlarged rietveld refined XRD patterns of 2θ between 41∼44.2° for [(Pr, Nd)_1−x_(La, Ce)_x_]_2.14_Fe_14_B powders. (**b**) Dependences of lattice parameters *a*, *c*, *c/a* ratio and unit cell volume *V* of the 2:14:1 tetragonal phase on the La-Ce content x.

As Ce^3+^ ion possesses a substantially larger radius (∼1.14 Å) than that of Ce^4+^ (∼0.97 Å), the appearance of Ce valence towards +3 state (carrying one 4f electron) is expected to be accompanied by an anomalous lattice expansion at the composition range of x > 0.3. Further step-scanned X-ray diffraction (XRD) patterns and the derived crystallographic parameters for [(Pr, Nd)_1−x_(La, Ce)_x_]_2.14_Fe_14_B (x = 0∼0.5) powders verify such an anomaly, as displayed in Figs 2, 3, 4. Figure 2 shows that 2:14:1 tetragonal phase is formed for all samples with the characteristic diffraction peaks corresponding to those of RE_2_Fe_14_B (space group *P4*_*2*_*/mnm*). To identify the structural changes and lattice parameters of 2:14:1 tetragonal phase in La-Ce co-doped specimen concretely, Rietveld refinements of experimental XRD profiles (black colors) have been performed. The optimized theoretical fits (red colors) and differences (blue colors) are also plotted. The difference pattern in each curve indicates a good matching between the calculated and experimental values. The refined structural parameters *a*, *c* and *V* (unit cell volume), and *R* factors are summarized in Table 1. Besides the matrix RE_2_Fe_14_B phase, small fractions of Fe and Nd phases (space group 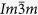 and *P6*_*3*_*/mmc*, respectively) are also identified. Meanwhile, for specimens with high La-Ce content (x ≥ 0.3), REFe_2_ phase (space group 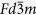) also appears, as verified by the appearance of additional diffraction peak (220) at 2θ ≈ 34.6° (Fig. 3a). Thermomagnetic characterizations for the sample with x = 0.3 in Fig. 3b further confirm the existence of REFe_2_ phase, whose Curie temperature corresponds to the observed phase transition peak at ∼229.1 K. Rietveld analysis in Fig. 2 also provides the detailed content of REFe_2_ phase (0.15, 0.23 and 0.09 wt.% for samples with x = 0.3, 0.4 and 0.5, respectively). Despite the appearance of minor impurities, La-Ce concentration in the 2:14:1 phase is rather close to the nominal composition, as characterized by EDS results (Table S1).

Figure 4a shows the enlarged XRD profiles with 2θ from 41 to 44.2°, illustrating the shift of those characteristic diffraction peaks of 2:14:1 phase with varied x. For example, (410) peak, as pointed out by dotted lines and arrows, firstly shifts to higher Bragg angle (0 ≤ x ≤ 0.3) and then turns to the lower side (0.3 < x ≤ 0.5), suggesting a non-linear dependence of lattice spacing on the La-Ce content x. The corresponding lattice parameters *a*, *c*, *a/c* and unit cell volume *V* for the tetragonal phase determined from the Rietveld refinements are plotted in Fig. 4b. *a* and *c* for (Pr, Nd)_2_Fe_14_B (x = 0) are 8.8096 Å and 12.2224 Å, respectively, in good agreement with the previously established results^9^. For the La-Ce co-doped samples, *a*, *c* and *V* do not linearly decrease or increase with higher La-Ce content. When x is below 0.3 (composition range I in Fig. 4b), lattice parameters decrease and can be approximately estimated by:


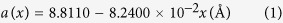



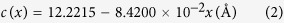



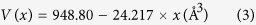


Further increasing La-Ce content to 0.4 and 0.5 (composition range II), *a*, *c* and *V* follow the opposite tendencies given by:


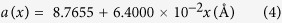



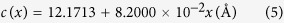



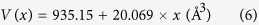


The linear reductions of *a* and *c* in composition range I (0 ≤ x ≤ 0.3) are commonly observed when Ce substitutes for Nd, following the empirical alloying theory. In composition range II (0.3 < x ≤ 0.5), however, the lattice parameters increase with growing La-Ce content, being consistent with the observed shift of Ce valence towards the +3 configuration in Fig. 1b. Besides, as demonstrated in Fig. S1, the electronic states of B/Fe/La/Nd remain unchanged with increasing La-Ce content, excluding their possible influences on the anomalous change of lattice parameters.

**Figure 5:**
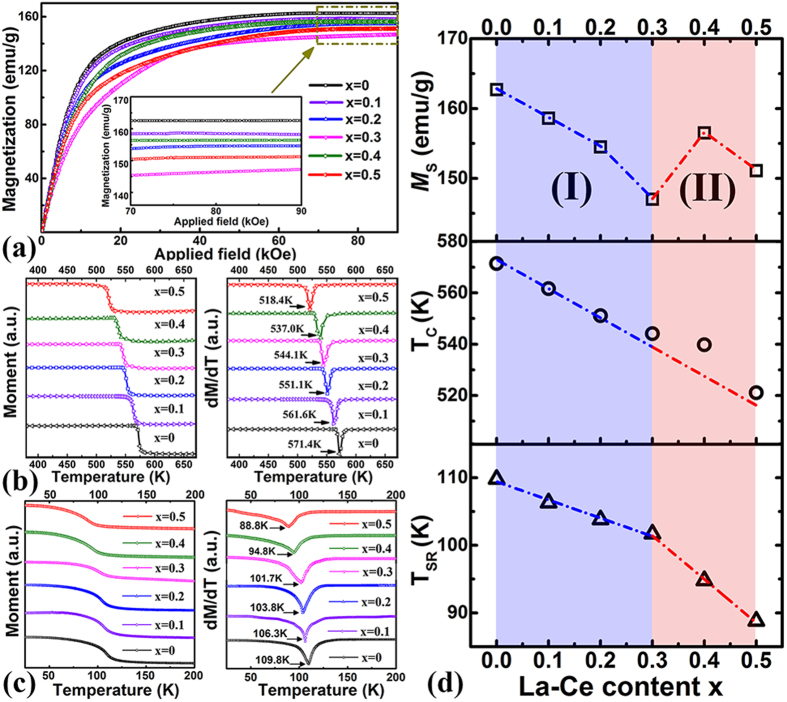
(**a**) Initial magnetization curves measured at 295 K, (**b**) *M*-*T* and d*M*/d*T*-*T* curves in the temperature range of 380~670 K, (**c**) *M*-*T* and d*M*/d*T*-*T* curves in the low temperature range of 25∼200 K for [(Pr, Nd)_1−x_(La, Ce)_x_]_2.14_Fe_14_B (x = 0∼0.5) samples. Inset in (**a**) is an enlarged view of the high-field regime. (**d**) The derived saturation magnetization *M*_s_, Curie temperature *T*_C_ and spin reorientation temperature *T*_SR_ as a function of La-Ce content x.

Figure 5a shows the initial magnetization curves of the [(Pr, Nd)_1−x_(La, Ce)_x_]_2.14_Fe_14_B strips at 295 K. The magnetization saturates at 90 kOe for all the samples, the value at which is then regarded as the saturation magnetization *M*_s_. In the composition range I (Fig. 5d), *M*_s_ decreases monotonically from 162.7 emu/g to 147.0 emu/g when x is increased from 0 to 0.3. On the contrary, in the composition range II, samples with x = 0.4 and 0.5 possess much larger *M*_s_ (156.5 and 151.1 emu/g, respectively). Though *M*_s_ of [(Pr, Nd)_0.5_(La, Ce)_0.5_]_2.14_Fe_14_B is smaller than that of [(Pr, Nd)_0.6_(La, Ce)_0.4_]_2.14_Fe_14_B due to the deteriorated interaction between the RE-Fe, it remains anomalously higher than the value for x = 0.3. Since the moment of REFe_2_ phase is smaller than that of the RE_2_Fe_14_B phase^29^, its appearance can only deteriorate the net magnetization. Figure 3b also indicates that REFe_2_ phase is paramagnetic at 295 K. Moreover, its fraction is quite small as revealed by the rietveld analysis in Fig. 2. Consequently, the abnormal increase in *M*_s_ for x = 0.4 and 0.5 cannot be attributed to the existence of secondary REFe_2_ phase. Meanwhile, characterizations on the Curie temperature *T*_C_ (Fig. 5b,d) also reveal a decreasing trend with increased La-Ce concentration, further excluding the effects of *T*_C_ on the abnormal magnetization enhancement at 295 K in the composition range II. Instead, it is resulted from the shift of Ce valence towards the +3 state (as indicated by the XPS spectra in Fig. 1) and the extra contribution of 4f electron. Further characterizations on the spin reorientation temperature *T*_SR_ (Fig. 5c,d) also show that *T*_SR_ diminishes with increased La-Ce content, and deviates from the linear fit of decrease with x = 0.3, 0.4 and 0.5. It suggests that the Ce valence change with one localized 4f moment also has an appreciable effect on lowering the spin reorientation temperature and retaining a [001] easy-axis alignment of magnetization in the low temperature range.

The above results have demonstrated that well-controlled La-Ce addition contributes to manipulating Ce valence towards the favorable +3 state. Besides the Ce valence, stable 2:14:1-type tetragonal structure also plays an indispensable role in affording high *M*_s_^9^. It should be noted that in terms of sole La substitution, unstable La_2_Fe_14_B phase tends to transform into α-Fe and La-B upon annealing at elevated temperatures in both as-cast and melt-spun La-Fe-B systems due to the large atomic radius of La^12^. Consequently, high substitution of La for Nd in the (Nd, La)_2_Fe_14_B compounds cannot be achieved as La prefers to enter into the grain boundary region^7^. However, in the present work of La-Ce co-doping, the *c/a* ratio keeps basically unchanged (Fig. 4b) despite of crystallographic anomalies in *a*, *c* and *V*. It suggests that increasing La-Ce substitution for Pr-Nd will not deteriorate the stability of tetragonal 2:14:1 structure even with x up to 0.5.

**Figure 6:**
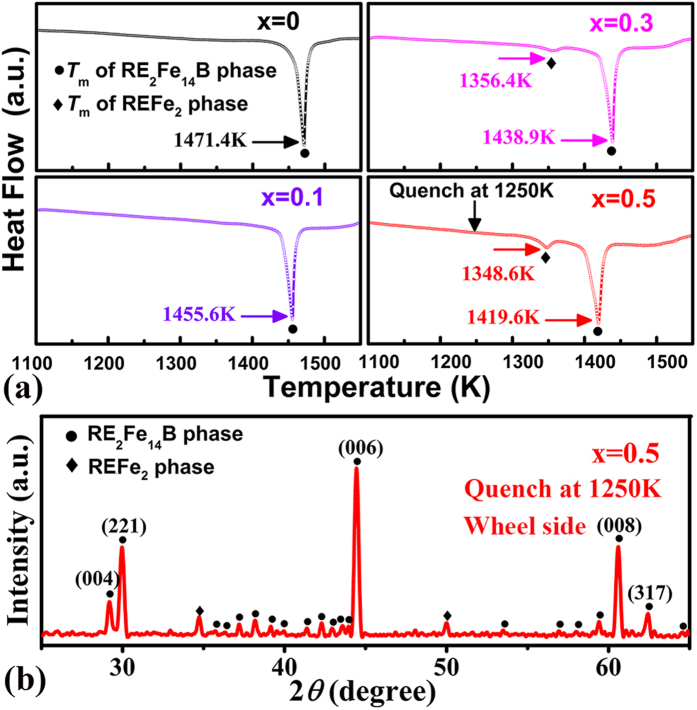
(**a**) DSC curves for specimens with x = 0, 0.1, 0.3, and 0.5 upon heating to 1550 K at 20 K/min, (**b**) XRD pattern of the as-quenched [(Pr, Nd)_0.5_(La, Ce)_0.5_]_2.14_Fe_14_B (x = 0.5) strip after annealing at 1250 K for 1 h.

To further investigate the stability of 2:14:1 phase, a thermal DSC analysis is carried out (upon heating to 1550 K as shown in Fig. 6a). An obvious endothermic peak is observed at 1471.4 K for the (Pr, Nd)_2.14_Fe_14_B specimen, which corresponds to the melting point of the RE_2_Fe_14_B phase^30^. Increasing La-Ce substitution for Pr-Nd lowers the melting point to 1455.6 K for x = 0.1, 1438.9 K for x = 0.3 and 1419.6 K for x = 0.5, respectively. When x is increased to 0.3 and 0.5, other relatively weak endothermic transitions are observed at 1356.4 K and 1348.6 K, respectively, which match the previously reported melting point of REFe_2_ phase^31^. Based on the thermal analysis, the strip with x = 0.5 was quenched into ice-water after annealing at 1250 K for 1 h to evaluate the high-temperature stability of the 2:14:1 phase. The XRD profile (Fig. 6b) on the wheel side of specimen shows that after quenching, the 2:14:1 matrix phase is stable. Minor REFe_2_ impurity also exists in this high La/Ce-containing specimen. Consequently, we can conclude that the 2:14:1-type tetragonal structure is well retained by La and Ce co-doping into the (Pr, Nd)_2.14_Fe_14_B compounds.

Previous research has shown that sole La substitution for Nd is beneficial to enlarge the unit cell size of 2:14:1 phase and Ce incorporation alone decreases the lattice^9^. In this study, La and Ce co-doping into the (Pr, Nd)_2.14_Fe_14_B compounds during induction melting, however, results in non-linear variation of lattice parameters with increasing La-Ce content x. At low La-Ce doping levels (x below 0.3), the reduced lattice constants are dominated by Ce addition. Afterwards, when the La-Ce content is above 0.3, the influence of La on expanding the unit cell increases (Fig. 4) and induces a Ce valence shift towards the +3 state (Fig. 1). Upon tuning the preferable Ce^3+^ valence, one 4f electron plays a positive role in enhancing the total magnetization as Ce is ferromagnetically coupled with Fe. Hence the magnetization measured in this work exhibits abnormal increment when x exceeds 0.3 (Fig. 5).

The finding that Ce valence can be manipulated by La-Ce co-doping may lead to a number of advantages. Firstly, high La-Ce substitution for Nd and excellent magnetic performance are generally recognized as contradictions for RE-Fe-B PMs due to the inferior intrinsic magnetic properties of La_2_Fe_14_B and Ce_2_Fe_14_B to Nd_2_Fe_14_B^9^. Our work, however, provides direct evidence that the Ce valence engineering via La-Ce co-doping is an effective approach to maintain the intrinsic magnetic properties, thus suppressing the magnetic dilution in La/Ce-containing RE_2_Fe_14_B system. Secondly, La-Ce co-doping provides a substantial possibility for developing high-performance RE-Fe-B magnets at significantly reduced material cost. As of February 2016, the cost of La-Ce alloy is approximately one-twelfth of Pr-Nd alloy (up-to-date RE cost is available at the website^32^), thus the total material cost can be lowered by about 57% with 50 at.% La-Ce replacement for Pr-Nd, e.g. $ 22 per kg for (Pr, Nd)_2_Fe_14_B versus $ 9.5 per kg for [(Pr, Nd)_0.5_(La, Ce)_0.5_]_2.14_Fe_14_B. In our on-going work, bulk (Pr, Nd, La, Ce)-Fe-B sintered magnets are prepared with La-Ce content as high as 50%. As shown in Fig. S2, sintered magnet with 50 at.% La-Ce co-substitution for Pr-Nd exhibits a much higher remanence *B*_r_ of 12.8 kGs, compared to those with single doping of La (12.2 kGs) or Ce (12.4 kGs) at the same concentration and processing routine. Thirdly, La-Ce co-substitution also provides a new recipe that the most abundant Ce and La can be utilized simultaneously in the hard magnets, contributing to the sustainable and balanced development of RE industry. Especially for La, which plays an indispensable role in inducing a favorable Ce valence shift and intrinsic magnetic properties accordingly. From the fundamental research view, it opens a new door to focus on the joint effect of multi rare earth substitution for those critical Nd/Pr/Dy/Tb.

In summary, it has been found that Ce valence shifts towards +3 configuration by co-doping La-Ce into (Pr, Nd)_2_Fe_14_B compounds when the doping level is above 0.3. This shifted valence with larger localized 4f moment is beneficial to strengthen the magnetization. Such an anomaly is ascribed to the expanded 2:14:1 phase lattice induced by the incorporation of La with larger atomic radius. Consequently, high La-Ce substitution for Pr-Nd allows the development of high-performance RE-Fe-B PMs at significantly reduced material cost and acts as a part of endeavor to the balanced utilization of RE sources.

## Methods

Alloys with the nominal composition of [(Pr, Nd)_1−x_(La, Ce)_x_]_2.14_Fe_14_B (x = 0, 0.1, 0.2, 0.3, 0.4 and 0.5) were prepared by induction melting and subsequent strip-casting at a copper wheel velocity of 1∼4 m/s, which is commonly used for mass production of Nd-Fe-B sintered magnets. The raw materials include high-purity (above 99.5%) La-Ce alloy (35 wt.% La-65 wt.% Ce), Pr-Nd alloy (20 wt.% Pr-80 wt.% Nd), Fe-B (81.5 wt.% Fe-18.5 wt.% B) alloy, and Fe metal. After grinding the strips, X-ray diffraction (XRD, SHIMADZU XRD-6000, Cu *K*_α_ radiation) patterns of the powders were recorded in 10° ≤ 2θ ≤ 100° angular range with a step of 0.01° and a counting time of 4s per step. Structural analysis was carried out with the Rietveld structural refinement program using Rietica software. Low temperature *M*-*T* curves (from 200 to 300 K, 200 Oe, 2 K/min) were measured using a superconducting quantum interference device (SQUID) to detect possible phase transitions. The chemical states of Ce/La/Nd/Fe/B were studied by means of X-ray photoelectron spectroscopy (XPS, Escalab 250Xi) after scraping the sample surface in high vacuum conditions. Room-temperature magnetization curves were measured by a Physical Property Measurement System (PPMS-9, Quantum Design) magnetometer up to 90 kOe. Curie temperature *T*_C_ and spin reorientation temperature *T*_SR_ of 2:14:1 phase were determined via the thermomagnetic curve in the temperature range of 380∼670 K and 25∼200 K, respectively, at 2 K/min with an external field of 200 Oe. Differential scanning calorimetric (DSC, NETZSCH TSA449) curves were measured upon heating to 1550 K at 20 K/min to determine the melting points of existing phases.

## Additional Information

**How to cite this article**: Jin, J. *et al.* Manipulating Ce Valence in RE_2_Fe_14_B Tetragonal Compounds by La-Ce Co-doping: Resultant Crystallographic and Magnetic Anomaly. *Sci. Rep.* **6**, 30194; doi: 10.1038/srep30194 (2016).

## Supplementary Material

Supplementary Information

## Figures and Tables

**Table 1:** Refined lattice parameters *a*, *c* and *V*, and *R* factors for the [(Pr, Nd)_1−x(_La, Ce)_x_]_2.14_Fe_14_B powders (x = 0∼0.5).

x	Lattice parameters			*R* factors		
	*a* (Å)	*c* (Å)	*V* (Å^3^)	*R*_p_	*R*_wp_	*R*_exp_
0	12.2224	8.8096	948.569	5.30	6.91	2.45
0.1	12.2120	8.8033	946.407	4.27	5.66	1.95
0.2	12.2040	8.7977	944.584	5.21	7.19	2.46
0.3	12.1970	8.7840	941.104	5.71	7.57	2.50
0.4	12.2020	8.7925	943.313	4.37	5.51	1.76
0.5	12.2134	8.7968	945.118	5.29	6.80	2.42
