# Supplementary material for: Manipulating Ce Valence in RE2Fe14B Tetragonal Compounds by La-Ce Co-doping: Resultant Crystallographic and Magnetic Anomaly
Source: Sci Rep. 2016 Jul 26;6:30194. doi: 10.1038/srep30194 (PMC4960486; doi:10.1038/srep30194)
Supplement: Supplementary Information [file srep30194-s1.doc]

Supplementary information

**Manipulating Ce Valence in RE_2_Fe_14_B Tetragonal Compounds by La-Ce Co-doping: Resultant Crystallographic and Magnetic Anomaly**

Jiaying Jin^1^, Yujing Zhang^1^, Guohua Bai^1^, Zeyu Qian^1^, Chen Wu^1^, Tianyu Ma^1,*^, Mi Yan^1,*^ & Baogen Shen^2^

^1^School of Materials Science and Engineering, State Key Laboratory of Silicon Materials, Key Laboratory of Novel Materials for Information Technology of Zhejiang Province, Zhejiang University, Hangzhou 310027, China

^2^State Key Laboratory for Magnetism, Institute of Physics, Chinese Academy of Sciences, Beijing 100190, China

E-mail: [maty@zju.edu.cn](mailto:jjy_zju@163.com); [mse_yanmi@zju.edu.cn](mailto:mse_yanmi@zju.edu.cn)


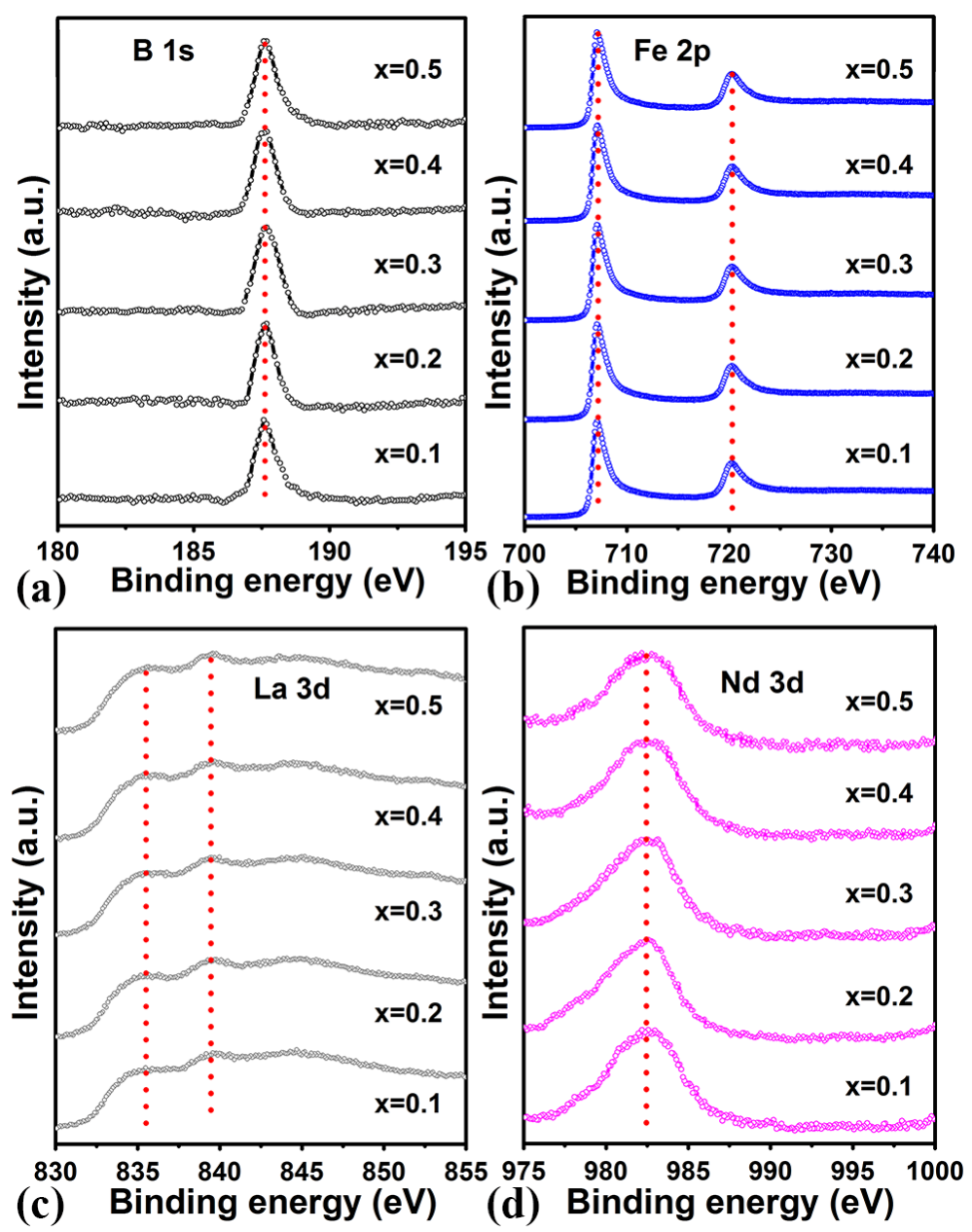


**Figure S1.** XPS spectra of (a) B 1s level, (b) Fe 2p level, (c) La 3d level, and (d) Nd 3d level in [(Pr, Nd)_1-x_(La, Ce)_x_]_2.14_Fe_14_B strips with x = 0.1~0.5.

In Fig. S1, the XPS spectra including B/Fe/La/Nd are displayed for [(Pr, Nd)_1-x_(La, Ce)_x_]_2.14_Fe_14_B strips with x = 0.1~0.5. It clearly reveals that differed La-Ce content x exerts no obvious influence on the ionic states of B/Fe/La/Nd. Only the Ce valence is changed towards the Ce^3+^ state (see Fig. 1), contributing to the lattice and magnetic anomalies of the 2:14:1 tetragonal phase.

*
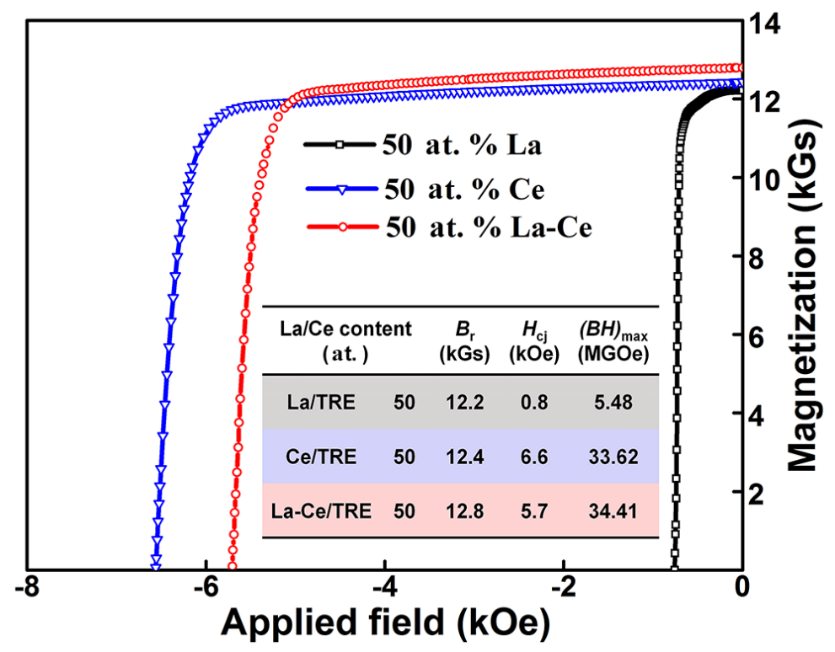
*

**Figure S2.** Demagnetization curves of sintered permanent magnets with 50 at. % La-Ce substitution, compared to those with La or Ce incorporation alone. The inset shows the detailed magnetic properties including *B*_r_, *H*_cj_, and *(BH)*_max_.

In Fig. S2, we provide further details on the hard magnetic properties of (La, Ce, Pr, Nd)-Fe-B PMs (red curve), in comparison to those with sole La (black curve) or Ce (blue curve) incorporation. The sintered magnet with 50 at. % La-Ce co-substitution exhibits a much superior remanence *B*_r_ of 12.8 kGs. In contrast, sole La or Ce incorporation at the same concentration leads to severe deterioration in *B*_r_ to 12.2 and 12.4 kGs, respectively.

**Table S1** EDS detections of La, Ce, Pr, Nd, Fe, and (La+Ce)/TRE content (at. %) from 2:14:1 matrix phase regions for [(Pr, Nd)_1-x_(La, Ce)_x_]_2.14_Fe_14_B strips with x = 0.1~0.5.

| **x** | **La** | **Ce** | **Pr** | **Nd** | **Fe** | **(La+Ce)/TRE** |
| --- | --- | --- | --- | --- | --- | --- |
| **0.1** | 0.51 | 0.95 | 2.57 | 10.18 | 85.79 | 0.103 |
| **0.2** | 0.98 | 1.76 | 2.20 | 8.74 | 86.32 | 0.200 |
| **0.3** | 1.46 | 2.74 | 1.92 | 7.74 | 86.14 | 0.303 |
| **0.4** | 1.97 | 3.64 | 1.67 | 6.69 | 86.03 | 0.402 |
| **0.5** | 2.36 | 4.46 | 1.39 | 5.37 | 86.42 | 0.502 |

To determine the detailed compositions of 2:14:1 matrix phase for specimens with x = 0.1~0.5, we performed EDS measurements in the back-scattered SEM images. The results are summarized in Table S1, showing the contents of La, Ce, Pr, Nd and Fe (it is difficult to obtain the precise boron content by EDS). Though REFe_2_ phase exists in the high La/Ce-containing specimens (x ≥ 0.3), the La-Ce concentration in the RE_2_Fe_14_B matrix phase is rather close to the nominal one, as revealed by the (La+Ce)/TRE content.
